# Novel 2-Thioxanthine and Dipyrimidopyridine Derivatives: Synthesis and Antimicrobial Activity †

**DOI:** 10.3390/molecules201019263

**Published:** 2015-10-22

**Authors:** Samar El-kalyoubi, Fatmah Agili, Shaker Youssif

**Affiliations:** 1 Organic Chemistry Department, Faculty of Pharmacy (Girls), ElAzhar University, Nasr City 11651, Egypt; 2 Chemistry Department, Faculty of Science, Jazan University, Jazan (Female Section) 45142, Saudi Arabia; E-Mail: dr.fatmah2000@gmail.com; 3 Medical Chemistry Division, Faculty of Medicine, Jazan University, Jazan 82621, Saudi Arabia; E-Mail: syoussif@hotmail.com; 4 Chemistry Department, Faculty of Science, Zagazig University, Zagazig 44511, Egypt

**Keywords:** 6-amino-2-thiouracil, 6-amino-5-benzylideneamino-2-thiouracil, 8-aryl-3-methyl-2-thioxanthines, 8-aryl-3-methyl-2-methylthiopurine-6-ones

## Abstract

Several fused imidazolopyrimidines were synthesized starting from 6-amino-1-methyl-2-thiouracil (**1**) followed by nitrosation, reduction and condensation with different aromatic aldehydes to give Schiff’s base. The dehydrocyclization of Schiff’s bases using iodine/DMF gave Compounds **5a**–**g**. The methylation of **5a**–**g** using a simple alkylating agent as dimethyl sulfate ((CH_3_)_2_SO_4_) gave either monoalkylated imidazolopyrimidine **6a**–**g** at room temperature or dialkylated derivatives **7a**–**g** on heating **6a**–**g** with ((CH_3_)_2_SO_4_). On the other hand, treatment of **1** with different aromatic aldehydes in absolute ethanol in the presence of conc. hydrochloric acid at room temperature and/or reflux with acetic acid afforded bis-5,5′-diuracylmethylene **8a**–**e**, which cyclized on heating with a mixture of acetic acid/HCl (1:1) to give **9a**–**e**. Compounds **9a**–**e** can be obtained directly by refluxing of Compound **1** with a mixture of acetic acid/HCl. The synthesized new compounds were screened for antimicrobial activity, and the MIC was measured.

## 1. Introduction

The importance of fused uracils, a common source for the development of new potential therapeutic agents [1,2], is well known. Fused uracils continue to attract considerable attention because of their great practical usefulness, primarily due to a very wide spectrum of biological activities. Uracils and their derivatives are considered to be important for drugs. A large number of uracil derivatives are reported to exhibit antimycobacterial [3], antitumor [4], antiviral [5] and anticancer [6,7] activities. In recent years, considerable attention has been focused on the development of new methodologies to synthesize many kinds of xanthine rings. Indeed, purines represent an important class of heterocyclic compounds having wide range of pharmaceutical and biological activities. The replacement of the oxygen by a sulfur atom may induce changes in the properties of the nucleobases and the ability to stabilize the DNA [8,9]. Furthermore, it has been suggested that the presence of the thio-group may enhance the probability of mutations that occur in DNA [10]. Therefore, versatile and widely-applicable methods for the synthesis of thiopurines are of considerable interest. On the other hand, acridine derivatives are known as antibacterial [11] and antitumor drugs [12,13] with clinical importance. The mechanism of the antibacterial action of acridine derivatives seems to be connected to the intercalation to DNA [14]. 1-Aza analogues of aminacrine and rivanol were, however, shown to be more active than the parent compounds against a hemolytic streptococcal strain [15], analogues of aminacrine and rivanol derived from 1:10-diazaanthracenes [16,17,18].

Our strategy has been directed towards the synthesis of a series of new thiouracil fused ring analogs, and their biological effects are determined.

## 2. Results and Discussion

### 2.1. Chemistry

**Scheme 1:**
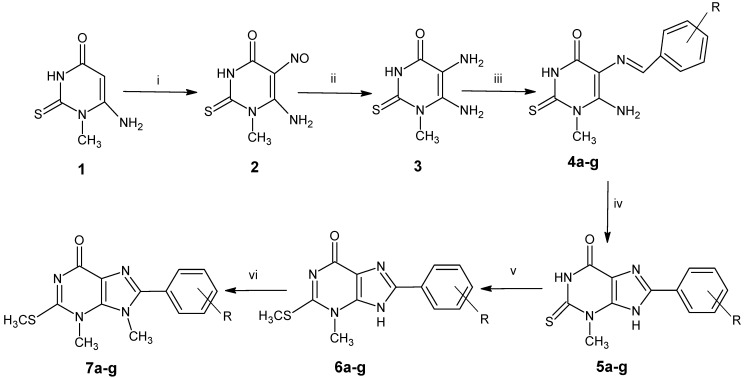
Synthesis of 8-aryl-3,9-dimethyl-2-methylthio-3,9-dihydro-6*H*-purin-6-one.

2-Thioxanthine [19] and 3-methyl-2-thioxanthine were previously prepared by different methods [20,21]. The alkylation of N-H or S-H takes place by many different reported methods in the literature [22,23]. Herein, we use one of the simplest methylating agents and a simple method. 8-Aryl-3-methyl-2-thioxanthines (**5a**–**g**) [21] were synthesized by the treatment of 6-amino-1-methyl-2-thiouracil (**1**) with sodium nitrite in acetic acid, affording 5-nitrosouracil **2**, followed by the reduction using ammonium sulfide, giving 4,5-diminouracil **3**. The obtained Compound **3** was condensed with different aromatic aldehydes in ethanol, affording 5-arylideneaminouracil **4a**–**g**, which undergoes dehydrocyclization via the reaction with I_2_/DMF, giving **5a**–**g** in a good yield. Methylation of **5a**–**g** takes place by the reaction with dimethyl sulfate in 0.5 N NaOH and ethanol for 1 h at room temperature with stirring to produce the target mono-alkylated thioxanthines **6a**–**g**; the methylation takes place smoothly on S-H (C-2). While refluxing of **6a**–**g** with dimethyl sulfate in 0.5 N NaOH and ethanol for 1 h gave **7a**–**g**, the methylation was carried out on N-H (C-9), as well as on the -OH group of the phenyl-8 in **7f**,**g** as shown in Scheme 1. The structures of the synthesized compounds were proven by ^1^H-NMR, elemental analysis, mass and IR spectra. ^1^H-NMR (DMSO-*d*_6_) for Compounds **6a**–**g** showed a characteristic signals at 4.0–4.54 ppm for S-CH_3_ and a characteristic signal at 10.02–11.11 ppm for N-H (**9**), while Compounds **7a**–**f** showed the disappearance of N-H (**9**) signals and the appearance of new N-CH_3_ at 3.38–3.79 ppm. Mass spectra of Compound **6b** showed fragmentation at M^+^ + 2 = 308, M^+^ = 306, and Compound **7b** showed M^+^ + 2 = 322, M^+^ = 320, due to the presence of the Cl atom attached to the phenyl group, as well as in compound **6d** showed fragmentation at M^+^ + 2 = 353, M^+^ = 351, and **7d** M^+^ + 2 = 367, M^+^ = 365, due to the presence of the Br atom; also, Ms of compounds **7f** showed M^+^ = 316, which explain the methylation of para OH of the phenyl group **8**.

**i** = NaNO_2_/AcOH/r.t.; **ii** = (NH_4_)_2_S; **iii** = ArCHO/EtOH; **iv** = I_2_/DMF; **v** = (CH_3_)_2_SO_4_/NaOH/EtOH/r.t; **vi** = (CH_3_)_2_SO_4_/NaOH/EtOH/reflux; where R in **6a**–**g** are: **a** = H; **b** = 4-Cl; **c** = 2-OCH_3_; **d** = 4-Br; **e** = 4-F; **f** = 4-OH; **g** = 2-OH; R in **7a**–**g** are: **a** = H; **b** = 4-Cl; **c** = 2-OCH_3_; **d** = 4-Br; **e** = 4-F; **f** = 4-OCH_3_; **g** = 2-OCH_3_.

**Scheme 2:**
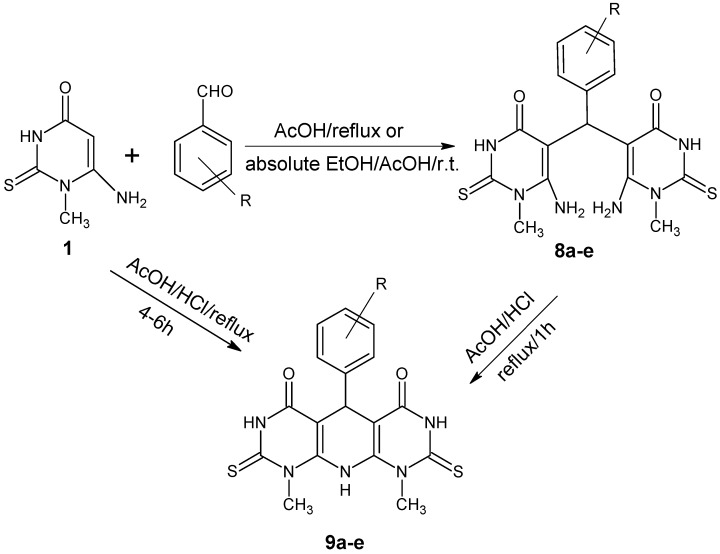
Reactions of aromatic aldehydes with 6-amino-1-methyl-2-thiouracil.

As part of our synthetic studies of fused uracils, we have reported the synthesis of dipyrimidopyridines [24,25]. Refluxing of Compound **1** with different aromatic aldehydes in acetic acid and/or absolute ethanol in the presence of conc. hydrochloric acid with stirring at room temperature produced the bis derivatives **8a**–**e**, which on refluxing compound **8a** for 1 h with a mixture of AcOH/HCl afforded the dipyrimidopyridine derivatives **9a**. Compounds **9a**–**e** can be synthesized directly in one step by refluxing Compound **1** with a mixture of AcOH/HCl for 4–6 h, as shown in Scheme 2. ^1^H-NMR (DMSO-*d*_6_) of Compounds **8a**–**e** showed a characteristic signal at 5.46–5.62 ppm for CH-5 and at 7.58–7.92 for NH_2_ (**6**), while ^1^H-NMR for Compounds **9a**–**e** showed a characteristic signals at 7.88–8.46 for NH-10 and at 6.06–6.22 for CH-5.

**Ar**: **a** = 4-Cl-C_6_H_4_; **b** = 3-NO_2_-C_6_H_4_; **c** = 4-NO_2_-C_6_H_4_; **d** = 4-Br-C_6_H_4_; **e** = 2-HO-C_6_H_4_.

### 2.2. Antimicrobial Screening

Results in Table 1 reveal that there is a vast variation between tested microorganisms, whereas *Escherichia coli* as a negative bacterial strains tolerate the applicable organic compounds. On the other hand, *S. aureus* is a positive bacterial strain affected by the application of Compounds **7d**, **8c** and **9a**. The most obvious inhibition zone was developed with Compound **7d** with the MIC reaching 0.4 µg followed by Compounds **9a** (MIC = 0.3 µg) and **8d** (the lowest effect). It is worth mentioning that the inhibitory effect induced by Compound **7d** was three-fold more than Compound **8d**. With respect to *C. albicans*, it is affected by Compounds **7b**, **7d**, **8c** and **9a** with the inhibition zone ranging between 8.3 and 16.0 mm. The highest inhibition was developed after application of Compound **9a** (16 mm) with the MIC reaching to 0.5 µg/mL.

## 3. Experimental Section

### 3.1. General

Melting points were determined with an Electro Thermal Mel-Temp II apparatus and are uncorrected. All reactions were monitored by thin layer chromatography (TLC) on pre-coated silica gel plates (0.25 mm, 20 × 20 cm, 60F_254_, E. Merck KGaA, Konstanz, Germany) with an appropriate solvent system ((A) 1:9 CH_3_OH–CHCl_3_; (B) 1:1 tolune–ethylacetate). IR spectra were obtained in the solid state in the form of KBr discs using a Perkin-Elmer Model 1430 spectrometer (Perkin-Elmer, Akron, OH, USA) and carried out in Taif University, Taif, KSA. ^1^H-NMR spectra were run at 400 MHz and ^13^C spectra were run at 125 MHz in dimethylsulfoxide (DMSO-*d*_6_) and TMS as an internal standard. Mass spectra were recorded on GC Ms-QP 5050A mass spectrometer (Shimadzu Corporation, Tokyo, Japan) at 70 eV and microanalytical data were performed on Elementar Vario EI III CHN analyzer (Elementar, Langenselbold, Germany) at the microanalytical unit, in Regional center for Mycology and Biotechnology, Al-Azhar University, Nasr City, Egypt.

**Table 1:** Detection of the antimicrobial activity of some organic compounds by measuring the formed inhibition zone (inhibition zone measured in mm and MIC in µg/mL).

Compd. No.	*Staphylococcus aureus*	*Escherichia coli*	*Candida albicans*
**6a**	0	0	0
**6b**	0	0	0
**6c**	0	0	0
**6d**	0	0	0
**6e**	0	0	0
**6f**	0	0	0
**6g**	0	0	0
**7a**	0	0	0
**7b**	0	0	8.3 ± 0.881
**7c**	0	0	0
**7d**	15.3 ± 0.88 * (0.4)	0	12.6 ± 1.1547 * (0.3)
**7e**	0	0	0
**7f**	0	0	0
**8a**	0	0	0
**8b**	0	0	0
**8c**	5.0 ±0.9 *	0	12.3 ± 0.91 * (0.3)
**8d**	0	0	0
**8e**	0	0	0
**9a**	14.6 ± 1.2 * (0.5)	0	16.0 ± 1.1547 * (0.5)
**9b**	0	0	0
**9c**	0	0	0
**9d**	0	0	0
**9e**	0	0	0
DMSO	0	0	0

* ±SE: standard error for three measurements; MIC: the value indicated between the brackets.

### 3.2. Synthesis of 8-Aryl-3-methyl-2-methylthio-3,9-dihydro-3,6H-purin-6-one *(**6a**–**g**)*

Dimethyl sulfate (7.0 mL) was added to a mixture of NaOH (12.5 mL, 0.5 N) and 2-thioxanthines **5a**–**g** (5 mmol). Ethanol was added gradually till completely dissolving of 2-thioxanthines. The mixture was stirred for 1 h at room temperature; the formed precipitate was collected by filtration, dried in the oven and crystallized from DMF/ethanol.

*3-Methyl-2-(methylthio)-8-phenyl-3,9-dihydro-6H-purin-6-one* (**6a**). Yield: 70%; m.p. >300 °C; IR (KBr) ν_max_ (cm^−1^): 3425 (NH), 3055 (CH aromatic), 2921 (CH aliphatic), 1688 (C=O), 1617 (C=N), 1290 (NH); ^1^H-NMR (DMSO-*d*_6_): 11.11 (s, 1H, NH), 7.83–7.77 (m, 2H, arom.), 7.58–7.55 (m, 3H, arom.), 4.03 (s, 3H, SCH_3_), 3.83 (s, 3H, NCH_3_); ^13^C-NMR (DMSO-*d*_6_): δ = 16.4 (SCH_3_), 29.28 (NCH_3_), 108.24, 114.40, 122.78, 128.01, 136.68, 142.92 (C-S), 150.76, 154.15, 154.17 (C=O) ppm; MS: *m*/*z* (%) = M^+^, 272 (7), 256 (9), 213 (16), 82 (100); Anal. calcd. for C_13_H_12_N_4_OS (272.32): C, 57.34; H, 4.44; N, 20.57. Found: C, 57.49; H, 4.51; N, 20.72.

*8-(4-Chlorophenyl)-3-methyl-2-(methylthio)-3,9-dihydro-6H-purin-6-one* (**6b**). Yield: 78%; m.p. >300 °C; IR (KBr) ν_max_ (cm^−1^): 3473 (NH), 3013 (CH arom.), 2955 (CH aliph.),1687 (C=O), 1612 (C=N), 1265 (NH); ^1^H-NMR (DMSO-*d*_6_): 10.31 (s, 1H, NH), 7.84–7.61 (d, 2H, *J* = 8.7, arom.), 7.60–7.58 (d, 2H, *J* = 8.4, arom.), 4.54 (s, 3H, SCH_3_), 3.63 (s, 3H, NCH_3_); ^13^C-NMR (DMSO-*d*_6_): δ = 16.6 (SCH_3_), 29.26 (NCH_3_), 107.21, 113.41, 122.78, 128.01, 136.68, 141.90 (C-S), 149.88, 153.18, 153.89 (C=O) ppm; MS: *m*/*z* (%) = M^+^ +2, 308 (19), M^+^ + 1, 307 (15), M^+^, 306 (54), 171 (33), 111 (28), 68 (100). Anal. calcd. for C_13_H_11_ClN_4_OS (306.77): C, 50.90; H, 3.91; N, 18.26. Found: C, 51.08; H, 3.94; N, 18.47.

*8-(2-Methoxyphenyl)-3-methyl-2-(methylthio)-3,9-dihydro-6H-purin-6-one* (**6c**). Yield: 67%; m.p. >300 °C; IR (KBr) ν_max_ (cm^−1^): 3450 (NH), 3076 (CH arom.), 2928 and 2838 (CH aliph.), 1694 (C=O), 1629 (C=N), 1286 (NH); ^1^H-NMR (DMSO-*d*_6_): 10.76 (s, 1H, NH), 8.04–8.01 (d, 1H, *J* = 1.8, arom.), 7.50–7.50 (m, 1H, *J* = 1.2, arom.), 7.22–7.19 (d, 1H, *J* = 8.4, arom.), 7.12–7.07 (m, 1H, *J* = 7.5, arom.), 4.43(s, 3H, SCH_3_), 3.93 (s, 3H, OCH_3_), 3.84 (s, 3H, NCH_3_). MS: *m*/*z* (%) = M^+^, 302 (95), 257 (51), 134 (65), 68 (100). Anal. calcd. for C_14_H_14_N_4_O_2_S (302.35): C, 55.61; H, 4.67; N, 18.53. Found: C, 55.74; H, 4.74; N, 18.69.

*8-(4-Bromophenyl)-3-methyl-2-(methylthio)-3,9-dihydro-6H-purin-6-one* (**6d**). Yield: 78%; m.p. >300 °C; IR (KBr) ν_max_ (cm^−1^): 3446 (NH), 3080 (CH arom.), 2924 and 2853 (CH aliph.), 1697 (C=O), 1633 (C=N), 1208 (NH); ^1^H-NMR (DMSO-*d*_6_): 10.88 (s, 1H, NH), 7.89–7.87 (d, 2H, *J* = 8.4, arom.), 7.59–7.56 (d, 2H, *J* = 8.1, arom.), 4.43 (s, 3H, SCH_3_), 3.82 (s, 3H, NCH_3_).MS: *m*/*z* (%) = M^+^ + 2, 353 (6), M^+^, 351 (9), 336 (12), 68 (100); Anal. calcd. for C_13_H_11_BrN_4_OS (351.22): C, 44.46; H, 3.16; N, 15.95. Found: C, 44.62; H, 3.12; N, 16.14.

*8-(4-Fluorophenyl)-3-methyl-2-(methylthio)-3,9-dihydro-6H-purin-6-one* (**6e**). Yield: 64%; m.p. >300 °C; IR (KBr) ν_max_ (cm^−1^): 3526 (NH), 3082 (CH arom.), 2933 and 2827 (CH aliph.), 1684 (C=O), 1625 (C=N), 1216 (NH); ^1^H-NMR (DMSO-*d*_6_): 11.02 (s, 1H, NH), 7.69–7.54 (m, 2H, arom.), 7.30–7.25 (m, 2H, arom.), 4.14 (s, 3H, SCH_3_), 3.72 (s, 3H, NCH_3_); ^13^C-NMR (DMSO-*d*_6_): δ = 16.3 (SCH_3_), 29.23 (NCH_3_), 107.11, 113.42, 122.64, 127.89, 136.57, 141.48 (C-S), 149.83, 153.01, 154.23 (C=O) ppm; MS: *m*/*z* (%) = M^+^ + 1, 291 (16), M^+^, 290 (27), 122 (32), 68 (100); Anal. calcd. for C_13_H_11_FN_4_OS (290.31): C, 53.78; H, 3.82; N, 19.30. Found: C, 53.87; H, 3.89; N, 19.37.

*8-(4-Hydroxyphenyl)-3-methyl-2-(methylthio)-3,9-dihydro-6H-purin-6-one* (**6f**). Yield: 63%; m.p. >300 °C; IR (KBr) ν_max_ (cm^−1^): 3615 (OH), 3406 (NH), 3071 (CH arom.), 2938 and 2839 (CH aliph.), 1700 (C=O), 1602 (C=N), 1253 (NH); ^1^H-NMR (DMSO-*d*_6_): 13.23 (s, 1H, OH), 10.02 (s, 1H, NH), 7.66–6.64 (d, 2H, *J* = 4.2, arom.), 6.88–6.86 (d , 2H, *J* = 6.9, arom.), 4.21 (s, 3H, SCH_3_), 3.83 (s, 3H, NCH_3_). MS: *m*/*z* (%) = M^+^, 288 (34), 258 (7), 120 (23), 82 (100), 67 (60); Anal. calcd. for C_13_H_12_N_4_O_2_S (288.32): C, 54.15; H, 4.20; N, 19.43. Found: C, 54.29; H, 4.27; N, 19.66.

*8-(2-Hydroxyphenyl)-3-methyl-2-(methylthio)-3,9-dihydro-6H-purin-6-one* (**6g**). Yield: 58%; m.p. >300 °C; IR (KBr) ν_max_ (cm^−1^): 3632 (OH), 3400 (NH), 3062 (CH arom.), 2925 and 2829 (CH aliph.), 1688 (C=O), 1624 (C=N), 1251 (NH); ^1^H-NMR (DMSO-*d*_6_): 13.12 (s, 1H, OH), 10.98 (s, 1H, NH), 7.820–7.815 (d, 1H, *J* = 1.5, arom.), 7.15–7.01 (m, 2H, arom.), 6.87–6.80 (m, 1H, arom.), 4.29 (s, 3H, SCH_3_), 3.79 (s, 3H, NCH_3_); 16.21 (SCH_3_), 28.98 (NCH_3_), 107.10, 113.41, 122.55, 127.72, 128.17, 136.61, 141.32 (C-S), 149.83, 152.24, 154.13 (C=O) ppm; MS: *m*/*z* (%) = M^+^, 288 (100), 274 (33), 215 (74), 120 (69); Anal. calcd. for C_13_H_12_N_4_O_2_S (288.32): C, 54.15; H, 4.20; N, 19.43. Found: C, 54.21; H, 4.24; N, 19.62.

### 3.3. 8-Aryl-3,9-dimethyl-2-methylthio-3,9-dihydro-6H-purin-6-one *(**7a**–**g**)*

Dimethyl sulfate (7.0 mL) was added to a mixture of NaOH (12.5 mL, 0.5 N) and 2-thioxanthines **6a**–**g** (5 mmol), and ethanol was added gradually till 2-thioxanthines were completely dissolved. The mixture was heated under reflux for 1 h with stirring. The reaction mixture was evaporated under reduced pressure, and the residue was collected by filtration and crystallized from ethanol to afford Compounds **7a**–**g**.

*3,9-Dimethyl-2-(methylsulfanyl)-8-phenyl-3,9-dihydro-6H-purin-6-one* (**7a**). Yield: 78%; m.p. >300 °C; IR (KBr) ν_max_ (cm^−1^): 3059 (CH arom.), 2980 (CH aliph.),1687 (C=O), 1609 (C=N); ^1^H-NMR (DMSO-*d*_6_): 7.79–7.76 (m, 2H, arom.), 7.54–7.51 (m, 3H, arom.), 4.00 (s, 3H, SCH_3_), 3.83 (s, 3H, NCH_3_), 3.72 (s, 3H, NCH_3_); 16.18 (SCH_3_), 29.38 (NCH_3_), 44.18 (NCH_3_), 108.13, 113.27, 122.42, 127.89, 136.57, 141.48 (C-S), 149.83, 153.01, 154.21 (C=O) ppm; MS: *m*/*z* (%) = M^+^, 286 (67), 82 (100); Anal. calcd. for C_14_H_14_N_4_OS (286.35): C, 58.72; H, 4.93; N, 19.57. Found: C, 58.89; H, 4.98; N, 19.64.

*3,9-Dimethyl-2-(methylsulfanyl)-8-(4-chlorophenyl)-3,9-dihydro-6H-purin-6-one* (**7b**). Yield: 82%; m.p. >300 °C; IR (KBr) ν_max_ (cm^−1^): 3068 (CH arom.), 2970 (CH aliph.), 1676 (C=O), 1623 (C=N); ^1^H-NMR (DMSO-*d*_6_): 7.62–7.60 (d, 2H, arom.), 7.55–7.52 (d, 2H, arom.), 4.03 (s, 3H, SCH_3_), 3.80 (s, 3H, NCH_3_), 3.66 (s, 3H, NCH_3_); MS: *m*/*z* (%) = M^+^ + 2, 322 (13), M^+^, 320 (30), 307 (16), 86 (11), 68 (100); Anal. calcd. for C_14_H_13_ClN_4_OS (320.79): C, 52.42; H, 4.08; N, 17.46. Found: C, 52.49; H, 4.13; N, 17.59.

*3,9-Dimethyl-2-(methylsulfanyl)-8-(2-methoxyphenyl)-3,9-dihydro-6H-purin-6-one* (**7c**). Yield: 71%; m.p. >300 °C; IR (KBr) ν_max_ (cm^−1^): 3092 (CH arom.), 2838 (CH aliph.), 1699 (C=O), 1633 (C=N); ^1^H-NMR (DMSO-*d*_6_): 7.46–7.45 (m, 1H, arom.), 7.26–7.23 (m, 2H, arom.), 7.13–7.09 (m, 1H, arom.), 4.13 (s, 3H, SCH_3_), 3.93 (s, 3H, OCH_3_), 3.79 (s, 3H, NCH_3_), 3.70 (s, 3H, NCH_3_); 16.31 (SCH_3_), 29.27 (NCH_3_), 44.16 (NCH_3_), 52.01 (OCH_3_), 108.13, 111.29, 113.27, 122.42, 127.89, 128.13, 136.57, 141.48 (C-S), 149.83, 153.01, 154.21 (C=O) ppm; MS: *m*/*z* (%) = M^+^, 316 (21), 302 (100), 287 (13), 243 (12), 119 (8), 70 (11), 64 (12); Anal. calcd. for C_15_H_16_N_4_O_2_S (316.37): C, 56.94; H, 5.10; N, 17.71. Found: C, 57.21; H, 5.17; N, 17.83.

*3,9-Dimethyl-2-(methylsulfanyl)-8-(4-bromophenyl)-3,9-dihydro-6H-purin-6-one* (**7d**). Yield: 79%; m.p. >300 °C; IR (KBr) ν_max_ (cm^−1^): 3079 (CH arom.), 2953(CH aliph.), 2841 (CH aliph.), 1689 (C=O), 1617 (C=N); ^1^H-NMR (DMSO-*d*_6_): 8.06–8.02 (m, 2H, arom.), 7.69–7.67 (m, 2H, arom.), 4.41 (s, 3H, SCH_3_), 3.74 (s, 3H, NCH_3_), 3.39 (s, 3H, NCH_3_); MS: *m*/*z* (%) = M^+^ + 2, 367 (8), M^+^, 365 (12), 348 (22), 68 (100); Anal. calcd. for C_14_H_13_BrN_4_OS (365.24): C, 46.04; H, 3.59; N, 15.34. Found: C, 46.19; H, 3.66; N, 15.46.

*3,9-Dimethyl-2-(methylsulfanyl)-8-(4-fluorophenyl)-3,9-dihydro-6H-purin-6-one* (**7e**). Yield: 69%; m.p. >300 °C; IR (KBr) ν_max_ (cm^−1^): 3074 (CH arom.), 2921 (CH aliph.), 2827 (CH aliph.), 1693 (C=O), 1618 (C=N), 1257 (NH); ^1^H-NMR (DMSO-*d*_6_): 7.87–7.84 (m, 2H, arom.), 7.40–7.36 (m, 2H, arom.), 4.03 (s, 3H, SCH_3_), 3.78 (s, 3H, NCH_3_), 3.67 (s, 3H, NCH_3_); MS: *m*/*z* (%) = M^+^ + 1, 305 (6), M^+^, 304 (18), 171 (33), 122 (54), 68 (100); Anal. calcd. for C_14_H_13_FN_4_OS (304.34): C, 55.25; H, 4.31; N, 18.41. Found: C, 55.34; H, 4.34; N, 18.56.

*3,9-Dimethyl-8-(4-methoxyphenyl)-2-(methylsulfanyl)-3,9-dihydro-6H-purin-6-one* (**7f**). Yield: 72%; m.p. >300 °C; IR (KBr) ν_max_ (cm^−1^): 3654 (OH), 3076 (CH arom.), 2928 (CH aliph.), 2839 (CH aliph.), 1695 (C=O), 1619 (C=N); ^1^H-NMR (DMSO-*d*_6_): 7.97–7.94 (d, 2H, arom.), 6.93–6.91 (d , 2H, arom.), 4.30 (s, 3H, SCH_3_), 4.05 (s, 3H, OCH_3_), 3.77 (s, 3H, NCH_3_), 3.64 (s, 3H, NCH_3_); MS: *m*/*z* (%) = M^+^, 316 (18), 302 (56), 268 (100), 214 (24); Anal. calcd. for C_15_H_16_N_4_O_2_S (316.37): C, 56.94; H, 5.10; N, 17.71. Found: C, 57.21; H, 5.07; N, 17.62.

*3,9-Dimethyl-8-(2-methoxyphenyl)-2-(methylsulfanyl)-3,9-dihydro-6H-purin-6-one* (**7g**). Yield: 67%; m.p. >300 °C; IR (KBr) ν_max_ (cm^−1^): 3052 (CH arom.), 2830 (CH aliph.), 1674 (C=O), 1627 (C=N); ^1^H-NMR (DMSO-*d*_6_): 7.43–7.41 (m, 1H, arom.), 7.24–7.21 (m, 2H, arom.), 7.18–7.12 (m, 1H, arom.), 4.22 (s, 3H, SCH_3_), 3.81 (s, 3H, OCH_3_), 3.68 (s, 3H, NCH_3_), 3.69 (s, 3H, NCH_3_); MS: *m/z* (%) = M^+^, 316 (21), 302 (100), 287 (13); Anal. calcd. for C_15_H_16_N_4_O_2_S (316.37): C, 56.94; H, 5.10; N, 17.71. Found: C, 57.18; H, 5.13; N, 17.85.

### 3.4. Synthesis of 5,5′-Arylmethylenebis-(6-Amino-1-methyl-2-thiouracil) *(**8a**–**e**)*

A mixture of 6-amino-1-methyl-2-thiouracil (**1**) (0.5 g, 2.4 mmol) and appropriate aromatic aldehydes (2.4 mmol) in absolute ethanol (20 mL) in the presence of conc. Hydrochloric acid (1mL) was stirred at room temperature for 1.5 h and/or reflux with glacial acetic acid (5 mL) for 1 h. The formed precipitate was filtered, washed with ethanol and crystallized from DMF/ethanol (3:1) into colorless crystals.

*5,5'-[(4-Chlorophenyl)methylene]bis(6-amino-1-methyl-2-thioxo-2,3-dihydropyrimidin-4(1H)-one)* (**8a**). Yield: 95%; m.p. = 240–242 °C; IR (KBr) ν_max_ (cm^−1^): 3442, 3340 (NH_2_ and NH), 3064 (CH arom.), 2871 (CH aliph.), 1732, 1662 (C=O), 1598 (C=C), 1086, 1119 (C=S), 747 (C-Cl); ^1^H-NMR (DMSO-*d*_6_): 12.28 (s, 2H, 2NH), 7.59 (bs, 4H, 2NH_2_), 7.26–7.24 (d, *J* = 5.9, 2H, arom.), 7.15–7.13 (d, *J* = 9.2, 2H, arom.), 5.48 (s, 1H, CH-5), 3.77 (s, 6H, 2 CH_3_); ^13^C-NMR (DMSO-*d*_6_): δ = 28.4 (CH), 38.7 (CH_3_), 91.2, 111.9, 117.2, 126.1, 129.3, 131.3, 162.2 (C=O), 171.1 (C=S) ppm; MS: *m*/*z* (%) = M^+^ + 2, 439 (8), M^+^ + 1, 438 (15), M^+^, 437 (22), 156 (82), 124 (77), 88 (100); Anal. calcd. for C_17_H_17_ClN_6_O_2_S_2_ (436.93): C, 46.73; H, 3.92; N, 19.23. Found: C, 46.98; H, 3.98; N, 19.42

*5,5'-[(3-Nitrophenyl)methylene]bis(6-amino-1-methyl-2-thioxo-2,3-dihydropyrimidin-4(1H)-one)* (**8b**). Yield: 88%; m.p. = 258–260 °C; IR (KBr) ν_max_ (cm^−1^): 3406, 3335 (NH_2_ and NH), 3057 (CH arom.), 2833 (CH aliph.), 1731, 1690 (C=O), 1528 (C=N), 1128, 1081 (C=S); ^1^H-NMR (DMSO-*d*_6_): 12.34 (s, 2H, 2NH), 8.03–8.01 (d, 1H, arom.), 7.9 (d, 1H, arom.), 7.63–7.61 (bs, 4H, 2 NH_2_), 7.54–7.50 (m, 2H, arom.), 5.60 (s, 1H, CH-5), 3.79 (s, 6H, 2 CH_3_); MS: *m*/*z* (%) = M^+^, 447 (37), 358 (48), 325 (18), 286 (100); Anal. calcd. for C_17_H_17_N_7_O_4_S_2_ (447.49): C, 45.63; H, 3.83; N, 21.91. Found: C, 45.85; H, 3.88; N, 22.14.

*5,5'-[(4-Nitrophenyl)methylene]bis(6-amino-1-methyl-2-thioxo-2,3-dihydropyrimidin-4(1H)-one)* (**8c**). Yield: 90%; m.p. = 261–263 °C; IR (KBr) ν_max_ (cm^−1^): 3450, 3328 (NH_2_ and NH), 3051 (CH arom.), 2992 (CH aliph.), 1714, 1663 (C=O), 1563 (C=N), 1116, 1086 (C=S); ^1^H-NMR (DMSO-*d*_6_): 12.34 (s, 2H, 2NH), 8.09–8.06 (d, *J* = 5.8, 2H, arom), 7.61 (bs, 4H, 2 NH_2_), 7.43–7.41 (d, *J* = 8.8, 2H, arom.), 5.59 (s, 1H, CH-5), 3.78 (s, 6H, 2 CH_3_); ^13^C-NMR (DMSO-*d*_6_): δ = 28.6 (CH), 39.2 (CH_3_), 91.7, 112.1, 117.5, 126.7, 129.6, 131.4, 162.4 (C=O), 171.3 (C=S) ppm; MS: *m*/*z* (%) = M^+^ , 447 (3), 377 (26), 286 (16), 198 (14), 149 (57), 125 (100); Anal. calcd. for C_17_H_17_N_7_O_4_S_2_ (447.49): C, 45.63; H, 3.83; N, 21.91. Found: C, 45.91; H, 3.81; N, 22.12.

*5,5'-[(4-Bromophenyl)methylene]bis(6-amino-1-methyl-2-thioxo-2,3-dihydropyrimidin-4(1H)-one)* (**8d**). Yield: 93%; m.p. = 224–226 °C; IR (KBr) ν_max_ (cm^−1^): 3442, 3308 (NH_2_ and NH), 3060 (CH arom.), 2946 (CH aliph.), 1727 (C=O), 1569 (C=N), 1142, 1077 (C=S); ^1^H-NMR (DMSO-*d*_6_): 12.28 (s, 2H, 2NH), 7.58 (bs, 4H, 2NH_2_), 7.39–7.37 (d, *J* = 5.8, 2H, arom.), 7.09–7.07 (d, *J* = 9.1, 2H, arom.), 5.46 (s, 1H, CH-5), 3.77 (s, 6H, 2 CH_3_); ^13^C-NMR (DMSO-*d*_6_): δ = 28.3 (CH), 38.7 (CH_3_), 91.4, 111.8, 117.3, 126.0, 129.2, 131.2, 162.4 (C=O), 171.3 (C=S) ppm; MS: *m*/*z* (%) = M^+^ + 2, 483 (5), M^+^, 481 (14), 449 (84), 249 (60), 208 (100), 193 (97), 167 (60); Anal. calcd. for C_17_H_17_BrN_6_O_2_S_2_ (481.38): C, 42.42; H, 3.56; N, 17.46 Found: C, 42.53; H, 3.58; N, 17.63.

*5,5'-[(2-Hydroxyphenyl)methylene]bis(6-amino-1-methyl-2-thioxo-2,3-dihydro-pyrimidin-4(1H)-one)* (**8e**). Yield: 89%; m.p. = 248–250 °C; IR (KBr) ν_max_ (cm^−1^): 3628 (OH), 3443, 3315 (NH_2_ and NH), 3028 (CH arom.), 2946 (CH aliph.), 1632 and 1602 (C=O), 1482 (C=N), 1145, 1087 (C=S); ^1^H-NMR (DMSO-*d*_6_): 13.82 (s, 1H, OH), 12.54 (s, 2H, 2NH), 7.29 (bs, 4H, 2NH_2_), 7.24–7.21 (d, *J* = 6.0, 1H, arom.), 7.18–7.16 (d, *J* = 6.0, 1H, arom.), 7.13–7.11 (m, 2H, arom.), 5.62 (s, 1H, CH-5), 3.80 (s, 6H, 2 CH_3_); ^13^C-NMR (DMSO-*d*_6_): δ = 28.3 (CH), 39.1 (CH_3_), 91.2, 112.3, 117.6, 126.3, 128.3, 129.7, 131.3, 132.0, 162.2 (C=O), 171.1 (C=S) ppm; MS: *m*/*z* (%) = M^+^, 418 (1), 377 (5), 97 (12), 91 (100); Anal. calcd. for C_17_H_18_N_6_O_3_S_2_ (418.49): C, 48.79; H, 4.34; N, 20.08. Found: C, 48.88; H, 4.42; N, 20.32.

### 3.5. Synthesis of 5-Aryl-1,9-dimethyl-2,8-dithioxo-2,3,5,8,9,10-hexahydropyrimido[5',4':5,6]pyrido [2,3-d]pyrimidine-4,6(1H,7H)-diones *(**9a**–**e**)*

Two methods were used to synthesize the target Compounds **9a**–**e**:

- A mixture of acetic acid/HCl (1:1, 2mL) was added to 6-amino-1-methyl-2-thiouracil (**1**) (0.26 g, 1.2 mmol) and appropriate aromatic aldehydes (1.2 mmol). The mixture was heated under reflux for 4–6 h. After cooling, the formed precipitate was filtered, washed with ethanol and crystallized from DMF, affording **9a**–**e**.
- A mixture of 5,5′-arylmethylene bis(6-amino-1-methyl-2-thiouracil) (**8a**) (0.1 g, 0.22 mmol) and acetic acid/HCl (1:1, 2 mL) was heated under reflux for 1 h. After cooling, the formed precipitate was filtered, washed with ethanol and crystallized from DMF to afford **9a**.

*5-(4-Chlorophenyl)-1,9-dimethyl-2,8-dithioxo-2,3,5,8,9,10-hexahydropyrimido [5',4':5,6]pyrido[2,3-d]pyrimidine-4,6(1H,7H)-dione* (**9a**). Yield (method A): 84%, (method B): 67%; m.p. = 298–300 °C; reflux 4 h; IR (KBr) ν_max_ (cm^−1^): 3219 (NH), 3046 (CH arom.), 2818 (CH aliph.), 1704 (C=O), 1561 (C=N), 1103, 1052 (C=S); ^1^H-NMR (DMSO-*d*_6_): 12.58 (s, 1H, NH), 12.50 (s, 1H, NH), 8.32 (s, 1H, NH (10)), 8.15–8.13 (d, *J* = 5.9, 1H, arom.), 7.94–7.92 (d, *J* = 8.9, 1H, arom.), 7.69–7.67 (d, *J* = 6.0, 1H, arom.), 7.57–7.55 (d, *J* = 8.8, 1H, arom.), 6.08 (s, 1H, CH-5), 3.57 (s, 3H, NCH_3_), 3.52 (s, 3H, NCH_3_); ^13^C-NMR (DMSO-*d*_6_): δ = 33.46 (CH), 45.9 (CH_3_), 125.08, 127.9, 128.2, 128.9, 129.52, 131.20, 161.9 (C=O), 169.8 (C=S) ppm; MS: *m*/*z* (%) = M^+^ + 2, 422 (0.3), M^+^, 420 (1), 129 (19), 74 (39), 69 (100); Anal. calcd. for C_17_H_14_ClN_5_O_2_S_2_ (419.90): C, 48.63; H, 3.63; N, 16.68. Found: C, 49.02; H, 2.86; N, 17.01.

*1,9-Dimethyl-5-(3-nitrophenyl)-2,8-dithioxo-2,3,5,8,9,10-hexahydropyrimido [5',4':5,6]pyrido[2,3-d] pyrimidine-4,6(1H,7H)-dione* (**9b**). Yield (method A): 86%; m.p. >300 °C; reflux 5 h; IR (KBr) ν_max_ (cm^−1^): 3461 (NH), 3060 (CH arom.), 2856 (CH aliph.), 1729 (C=O), 1582 (C=N), 1166, 1092 (C=S); ^1^H-NMR (DMSO-*d*_6_): 12.56 (s, 1H, NH), 12.05 (s, 1H, NH), 8.15 (s, 1H, NH(10)), 8.06 (s, 1H, arom.), 7.96–7.48 (m, 3H, arom.), 6.22 (s, 1H, CH-5), 3.58 (s, 3H, NCH_3_), 3.52 (s, 3H, NCH_3_); ^13^C-NMR (DMSO-*d*_6_): δ = 33.56 (CH), 46.33 (CH_3_), 125.24, 126.72, 127.45, 128.12, 128.43, 129.21, 129.91, 131.82, 162.4 (C=O), 171.3 (C=S) ppm; MS: *m*/*z* (%) = M^+^, 430 (5), 428 (16), 299 (18), 106 (100); Anal. calcd. for C_17_H_14_N_6_O_4_S_2_ (430.46): C, 47.43; H, 3.28; N, 19.52. Found: C, 47.94; H, 2.85; N, 19.97.

*1,9-Dimethyl-5-(4-nitrophenyl)-2,8-dithioxo-2,3,5,8,9,10-hexahydropyrimido [5',4':5,6]pyrido[2,3-d] pyrimidine-4,6(1H,7H)-dione* (**9c**). Yield (method A): 83%; m.p. >300 °C; reflux 4 h, IR (KBr) ν_max_ (cm^−1^): 3384 (NH), 3034 (CH arom.), 2918 (CH aliph.), 1705 (C=O), 1520 (C=N), 1134, 1086 (C=S); ^1^H-NMR (DMSO-*d*_6_): 11.93 (s, 2H, 2NH), 8.46 (s, 1H, NH(10)), 8.06–8.04 (s, *J* = 7.5, 2H, arom.), 7.29–7.27 (s, *J* = 7.5, 2H, arom.), 6.20 (s, 1H, CH-5), 3.51 (s, 6H, 2 NCH_3_); ^13^C-NMR (DMSO-*d*_6_): δ = 33.55 (CH), 46.41 (CH_3_), 125.27, 128.11, 128.43, 128.93, 129.86, 131.34, 161.93 (C=O), 169.78 (C=S) ppm; MS: *m*/*z* (%) = M^+^, 430 (1), 332 (24), 106 (68), 91 (14), 78 (100); Anal. calcd. for C_17_H_14_N_6_O_4_S_2_ (430.46): C, 47.43; H, 3.28; N, 19.52. Found: C, 48.01; H, 2.86; N, 19.93.

*5-(4-Bromophenyl)-1,9-dimethyl-2,8-dithioxo-2,3,5,8,9,10-hexahydropyrimido [5',4':5,6]pyrido[2,3-d] pyrimidine-4,6(1H,7H)-dione* (**9d**). Yield (method A): 90%; m.p. >300 °C; reflux 4 h; IR (KBr) ν_max_ (cm^−1^): 3220 (NH), 3046 (CH arom.), 2818 (CH aliph.), 1721 (C=O), 1567 (C=N), 1107, 1068 (C=S); ^1^H-NMR (DMSO-*d*_6_): 12.61 (s, 1H, NH), 12.52 (s, 1H, NH), 8.28–8.26 (d, 1H, arom.), 8.05–8.03 (d, *J* = 8.6, 1H, arom.), 7.99–7.97 (d, *J* = 8.8, 2H, arom.), 7.84 (s, 1H, NH(10)), 6.06 (s, 1H, CH-5), 3.56 (s, 3H, NCH_3_), 3.51 (s, 3H, NCH_3_); ^13^C-NMR (DMSO-*d*_6_): δ = 33.48 (CH), 45.88 (CH_3_), 125.16, 127.89, 128.16, 128.91, 129.47, 131.16, 161.89 (C=O), 169.77 (C=S) ppm; MS: *m*/*z* (%) = M^+^ + 2, 466 (2), M^+^, 464 (6), 200 (58), 95 (100); Anal. calcd. for C_17_H_14_BrN_5_O_2_S_2_ (464.35): C, 43.97; H, 3.04; N, 15.08 Found: C, 44.34; H, 2.60; N, 15.31.

*5-(2-Hydroxyphenyl)-1,9-dimethyl-2,8-dithioxo-2,3,5,8,9,10-hexahydropyrimido [5',4':5,6]pyrido[2,3-d]pyrimidine-4,6(1H,7H)-dione* (**9e**). Yield (method A): 81%; m.p. >300 °C; reflux 6 h; IR (KBr) ν_max_ (cm^−1^): 3321 (NH), 3096 (CH arom.), 2901 (CH aliph.), 1684 (C=O), 1580 (C=N), 1141, 1081 (C=S); ^1^H-NMR (DMSO-*d*_6_): 12.44 (s, 1H, OH), 12.36 (s, 1H, NH), 10.96 (s, 1H, NH), 8.40–8.38 (d, 1H, arom.), 8.36–8.34 (d, 1H, arom.), 8.28–8.26 (d, 1H, arom.), 7.88 (s, 1H, NH(10)), 6.91–6.89 (d, 1H, arom.), 6.17 (s, 1H, CH-5), 3.56 (s, 6H, 2 NCH_3_); ^13^C-NMR (DMSO-*d*_6_): δ = 33.46 (CH), 45.64 (CH_3_), 125.02, 127.84, 128.12, 128.76, 129.32, 131.14, 161.85 (C=O), 169.73 (C=S) ppm; MS: *m*/*z* (%) = M^+^, 401 (0.4), 109 (36), 106 (100), 78 (51), 43 (52); Anal. calcd. for C_17_H_15_N_5_O_3_S_2_ (401.46): C, 50.86; H, 3.77; N, 17.44. Found: C, 51.27; H, 3.31; N, 17.70.

## 4. Antimicrobial Activity

### 4.1. Microorganisms and Culture Media

*Staphylococcus aureus* (Gram-positive), *Escherichia coli* (Gram-negative) and *Candida albicans* (yeast) were obtained from the microbiology lab of the Faculty of Medicine, Jazan University Culture Collection. All bacteria strains were maintained and kept at −80 °C until used. Müller–Hinton agar (MHA) and Sabouraud dextrose agar (SDA) were obtained from the microbiology lab of the Faculty of Medicine, Jazan University.

### 4.2. Disc Diffusion Method

The disc diffusion method for antimicrobial susceptibility testing was carried out according to the standard method [26] to assess the presence of antibacterial activities of the tested chemical substances. A microbial culture (which has been adjusted to 0.5 McFarland standards) was used to inoculate Müller–Hinton agar and Sabouraud dextrose agar plates evenly using a sterile swab. The plates were dried for 15 min and then used for the sensitivity test. The discs that had been impregnated with 1 mg/mL of the chemical samples were placed on the Müller–Hinton agar and Sabouraud dextrose agar surface. Each test plate comprises six discs: one negative control (disc with the used solvent) and five treated discs. The negative control was DMSO (100%). Besides the controls, each plate had five treated discs were placed about equidistant from each other. The plate was then incubated at 37 °C for 18–24 h depending on the species of bacteria or yeast used in the test. After the incubation, the plates were examined for the inhibition zone. The inhibition zone was then measured using calipers and recorded. The test was performed in triplicates to ensure reliability.

### 4.3. Minimum Inhibition Concentration Determination

Minimum inhibition concentration determination was performed according to the method described in [27,28,29]. Serial dilutions of the chemical substances that showed antimicrobial activity with the disc diffusion method (a reasonable inhibition zone) (a range of 10 dilutions from 0.1 µg/mL up to 1 µg/mL) are added to a Müller–Hinton or and Sabouraud dextrose broth medium in separate test tubes. These tubes are then inoculated with the microorganism that one wishes to test (*Staphylococcus aureus*, *Escherichia coli* or *Candida albicans)*. The tubes are allowed to incubate overnight. Broth tubes that appear turbid are indicative of bacterial growth, while tubes that remain clear indicate no growth. The MIC of the antibiotic is the lowest concentration that does not show growth.

## 5. Conclusions

Simple methylation methods were used for the methylation of S-H and N-H in the 2-thioxanthines. Also, the formation of tricyclic dipyrimidopyridines was developed. Compounds **7d** and **9a** showed a significant effect against *Staphylococcus aureus* and *Candida albicans*.

## References

[1] Wamhoff H., Dzenis J., Hirota K. (1992). Uracils: Versatile Starting Materials in Heterocyclic Synthesis. Adv. Heterocycl. Chem..

[2] González-Vallinas M., Molina S., Vicente G., Cueva A., Vargas T., Santoyo S., García-Risco M.R., Fornari T., Reglero G., Molina A.R. (2013). Antitumor effect of 5-fluorouracil is enhanced by rosemary extract in both drug sensitive and resistant colon cancer cells. Pharmacol. Res..

[3] Kumar A., Sinha S., Chauhan P.M. (2002). Syntheses of novel antimycobacterial combinatorial libraries of structurally diverse substituted pyrimidines by three-component solid-phase reactions. Bioorg. Med. Chem. Lett..

[4] Baraldi P.G., Pavani M.G., Nunez M., Nuñez M.C., Brigidi P., Vitali B., Gambari R., Romagnoli R. (2002). Antimicrobial and antitumor activity of *N*-heteroimmine-1,2,3-dithiazoles and their transformation in triazolo-, imidazo-, and pyrazolopirimidines. Bioorg. Med. Chem..

[5] Nasr M.N., Gineinah M.M. (2002). Pyrido[2,3-*d*]pyrimidines and pyrimido[5′,4′:5,6]pyrido[2,3-*d*]pyrimidines as new antiviral agents: Synthesis and biological activity. Arch. Pharm..

[6] Nagarapu L., Vanaparthi S., Bantu V., Kumar C.G. (2013). Synthesis of Novel Benzo[4,5]thiazolo[1,2-*a*]pyrimi-dine-3-carboxylate Derivatives and Biological Evaluation as Potential Anticancer Agents. Eur. J. Med. Chem..

[7] Sondhi S.M., Johar M., Rajvanshi S., Dastidar S.G., Shukla R., Raghubir R., Lown J.W. (2001). Anticancer, anti-inflammatory and analgesic activity evaluation of heterocyclic compounds synthesized by the reaction of 4-isothiothiocyanato-4-methylpentan-2-one with substituted *o*-phenylenediamines, *o*-diaminopyridine and (un)substituted *o*-diaminopyrimidines. Aust. J. Chem..

[8] Saenger W. (1984). Principles of Nucleic Acid Structure.

[9] Ono M., Kawakami M. (1977). Separation of newly-synthesized RNA by organomercurial agarose affinity chromatography. J. Biochem..

[10] Leszczynski J. (1993). Tautomers of 6-thioguanine: Structures and properties. J. Phys. Chem..

[11] Nagdi L., Galy J.P., Barbe J., Cremieux A., Chevalier J., Sharples D. (1990). Some new 1-nitro acridine derivatives as antimicrobial agents. Eur. J. Med. Chem..

[12] Pawlak K., Pawlak J.W., Konopa J. (1984). Cytotoxic and antitumor activity of 1-nitroacridines as an aftereffect of their interstrand DNA cross-linking. Cancer Res..

[13] Gunaratnam M., Green C., Moreira J.B., Moorhouse A.D., Kelland L.R., Moses J.E., Neidle S. (2009). G-quadruplex compounds and cis-platin act synergistically to inhibit cancer cell growth *in vitro* and *in vivo*. Biochem. Pharmacol..

[14] Nesměrák K., Pospíšek M., Zikánová B., Němec I., Barbe J., Gabriel J. (2002). Effect of structure on antibiotic action of new 9-(ethylthio)acridines. Folia Microbiol..

[15] Besly D.M., Goldberg A.A. (1957). Potential antimalarial derivatives of triaza-anthracene. J. Chem. Soc..

[16] Bruce-Chwatt L.J., Archibald H.M. (1953). Field trials of new antimalarials in West Africa. Br. Med. J..

[17] Greenwood D. (1995). Conflicts of interest: The genesis of synthetic antimalarial agents in peace and war. J. Antimicrob. Chemother..

[18] Wainwright M., Phoenix D.A., Marland J., Wareing D.R., Bolton F.J. (1997). *In-vitro* photobactericidal activity of aminoacridines. J. Antimicrob. Chemother.

[19] Robins R.K., Dille K.J., Willits C.H., Christensen B.E. (1953). Purines. II. The Synthesis of Certain Purines and the Cyclization of Several Substituted 4,5-Diaminopyrimidines. J. Am. Chem. Soc..

[20] Bergmann F., Levin G., Kalmus A., Kwietny-Govrin H. (1961). Synthesis and Properties of 3-Methylpurines. J. Org. Chem..

[21] Youssif S., Agili F., Mohamed S.F. (2009). Syntheis, Antiviral and Antimicrobial activities of New Substituted Thioxanthines and Thiolumazines. J. Appl. Sci. Res..

[22] Stanovnik B., Tisler M., Hribar A., Barlin G.B., Brown D.J. (1981). Methylation of heterocyclic compounds containing NH, SH and/or OH groups by means of *N*,*N*-dimethylformamide dimethyl acetal. Aust. J. Chem..

[23] Lamoureux G., Agüero C. (2009). A comparison of several modern alkylating agents. Arkivoc.

[24] Youssif S., El-Bahaie S., Nabih E. (2003). A Facile One-pot Synthesis of Fused 2-Thiouracils: Dipyrimidinopyridine, Pyrazolopyrimidine and Pyridazinopyrimidines. Bull. Korean Chem. Soc..

[25] Youssif S., Mohamed S.F. (2008). 6-Amino-2-thio- and 6-Aminouracils as Precursors for the Synthesis of Antiviral and Antimicrobial Methylenebis(2-thiouracils), Tricyclic Pyrimidines, and 6-Alkylthiopurine-2-ones. Chem. Mon..

[26] Bauer A.W., Kirby W.M., Sherris J.C., Turck M. (1966). Antibiotic susceptibility testing by a standardized single disk method. Am. J. Clin. Pathol..

[27] Wiegand I., Hilpert K., Hancok R.E. (2008). Agar and broth dilution methods to determine the minimal inhibitory concentration (MIC) of antimicrobial substances. Nat. Protoc..

[28] Hammer K.A., Carson C.F., Riley T.V. (1996). Susceptibility of transient and commensal skin flora to the essential oil of *Melaleuca alternifolia* (tea tree oil). Am. J. Infect. Control.

[29] Turnidge J.D., Ferraro M.J., Jorgensen J.H., Murray P.R., Baron E.J., Jorgensen J.H., Pfaller M.A., Yolken R.H. (2003). Susceptibility Test Methods. General Considerations. Manual of Clinical Microbiology.

