# Correction: El-kalyoubi, S.; et al. Novel 2-Thioxanthine and Dipyrimidopyridine Derivatives: Synthesis and Antimicrobial Activity. *Molecules*, 2015, *20*, 19263--19276

**DOI:** 10.3390/molecules23071592

**Published:** 2018-06-29

**Authors:** Samar El-kalyoubi, Fatmah Agili, Shaker Youssif

**Affiliations:** 1 Organic Chemistry Department, Faculty of Pharmacy (Girls), Al-Azhar University, Nasr City 11651, Egypt; 2 Chemistry Department, Faculty of Science, Jazan University, Jazan (Female Section) 45142, Saudi Arabia; dr.fatmah2000@gmail.com; 3 Medical Chemistry Division, Faculty of Medicine, Jazan University, Jazan 82621, Saudi Arabia; syoussif@hotmail.com; 4 Chemistry Department, Faculty of Science, Zagazig University, Zagazig 44511, Egypt

The authors wish to make the following changes to their paper [1]. The omitted to put ^13^C-NMR before the data of three compounds. Also, they repeated the same IR and ^13^C-NMR data for one compound with the same structure, as well as some minor typing errors. Please note the following:

1. On page 19264, line 31, the sentence “giving 4,5-diminouracil 3” should be replaced with “giving 4,5-diaminouracil 3”.
2. On page 19268, line 11, the sentence “Anal. calcd. for C_13_H_11_ClN_4_OS (306.77): C, 50.90; H, 3.91; N, 18.26” should be replaced with “Anal. calcd. for C_13_H_11_ClN_4_OS (306.77): C, 50.90; H, 3.61; N, 18.26”.
3. On page 19268, lines 14 and 15, the sentence “^1^H-NMR (DMSO-*d*_6_): 10.76 (s, 1H, NH), 8.04–8.01 (d, 1H, *J* = 1.8, arom.), 7.50–7.50 (m, 1H, *J* = 1.2, arom.), 7.22–7.19 (d, 1H, *J* = 8.4, arom.), 7.12–7.07 (m, 1H, *J* = 7.5, arom.)……” should be replaced with “^1^H-NMR (DMSO-*d*_6_): 10.76 (s, 1H, NH), 8.04–8.01 (d, 1H, *J* = 1.8, arom.), 7.51–7.50 (m, 1H, arom.), 7.22–7.19 (d, 1H, *J* = 8.4, arom.), 7.12–7.07 (m, 1H, *J* = 7.5, arom.)……”.
4. On page 19268, line 29, the sentence “107.11, 113.42, 122.64, 127.89, 136.57, 141.48 (C-S), 149.83, 153.01, 154.23 (C=O) ppm” should be replaced with “107.11, 113.42, 122.64, 127.89, 141.48 (C-S), 146.57, 149.83, 153.01, 154.23 (C=O) ppm”.
5. On page 19268, line 34, the sentence “^1^H-NMR (DMSO-*d*_6_): 13.23 (s, 1H, OH), 10.02 (s, 1H, NH), 7.66–6.64” should be replaced with “^1^H-NMR (DMSO-*d*_6_): 13.23 (s, 1H, OH), 10.02 (s, 1H, NH), 7.66–7.65”.
6. On page 19269, lines 3–5, the sentence “^1^H-NMR (DMSO-*d*_6_): 13.12 (s, 1H, OH), 10.98 (s, 1H, NH), 7.820–7.815 (d, 1H, *J* = 1.5, arom.), 7.15–7.01 (m, 2H, arom.), 6.87–6.80 (m, 1H, arom.), 4.29 (s, 3H, SCH_3_), 3.79 (s, 3H, NCH_3_); 16.21 (SCH_3_), 28.98 (NCH_3_), 107.10, 113.41, 122.55, 127.72, 128.17, 136.61, 141.32 (C-S), 149.83, 152.24, 154.13 (C=O) ppm” should be replaced with “^1^H-NMR (DMSO-*d*_6_): 13.12 (s, 1H, OH), 10.98 (s, 1H, NH), 7.82–7.81 (d, 1H, *J* = 1.5, arom.), 7.15–7.01 (m, 2H, arom.), 6.87–6.80 (m, 1H, arom.), 4.29 (s, 3H, SCH_3_), 3.79 (s, 3H, NCH_3_); ^13^C-NMR (DMSO-*d*_6_): δ = 16.21 (SCH_3_), 28.98 (NCH_3_), 107.10, 113.41, 122.55, 127.72, 128.17, 136.61, 139.29, 141.32 (C-S), 149.83, 152.24, 154.13 (C=O) ppm”.
7. On page 19269, line 17, the sentence “16.18 (SCH_3_), 29.38 (NCH_3_), 44.18 (NCH_3_), 108.13, 113.27” should be replaced with “^13^C-NMR (DMSO-*d*_6_): δ = 16.18 (SCH_3_), 29.38 (NCH_3_), 44.18 (NCH_3_), 108.13, 113.27”.
8. On page 19269, lines 29–31, the sentence “16.31 (SCH_3_), 29.27 (NCH_3_), 44.16 (NCH_3_), 52.01 (OCH_3_), 108.13, 111.29, 113.27, 122.42, 127.89, 128.13, 136.57, 141.48 (C-S), 149.83, 153.01, 154.21 (C=O) ppm” should be replaced with “^13^C-NMR (DMSO-*d*_6_): δ = 16.31 (SCH_3_), 29.27 (NCH_3_), 44.16 (NCH_3_), 52.01 (OCH_3_), 111.29, 118.23, 123.27, 124.42, 127.89, 128.13, 139.51, 141.58 (C-S), 152.83, 153.81, 154.41 (C=O) ppm”.
9. On page 19270, line 10, the sentence “IR (KBr) ν_max_ (cm^−1^): 3654 (OH), 3076 (CH arom.), 2928 (CH aliph.), 2839 (CH aliph.)” should be replaced with “IR (KBr) ν_max_ (cm^−1^): 3076 (CH arom.), 2928 (CH aliph.), 2839 (CH aliph.)”.
10. On page 19270, lines 16–19, the sentence “IR (KBr) ν_max_ (cm^−1^): 3052 (CH arom.), 2830 (CH aliph.), 1674 (C=O), 1627 (C=N); ^1^H-NMR (DMSO-*d*_6_): 7.43–7.41 (m, 1H, arom.), 7.24–7.21 (m, 2H, arom.), 7.18–7.12 (m, 1H, arom.), 4.22 (s, 3H, SCH_3_), 3.81 (s, 3H, OCH_3_), 3.68 (s, 3H, NCH_3_), 3.69 (s, 3H, NCH_3_); MS: *m*/*z* (%) = M^+^, 316 (21), 302 (100), 287 (13)” should be deleted.
11. On page 19270, line 31, the sentence “91.2, 111.9, 117.2, 126.1, 129.3, 131.3, 162.2 (C=O), 171.1 (C=S) ppm; MS: *m*/*z* (%) = M^+^ + 2” should be replaced with “91.2, 111.9, 117.2, 126.1, 129.3, 151.3, 162.2 (C=O), 171.1 (C=S) ppm; MS: *m*/*z* (%) = M^+^ + 2”.
12. On page 19271, line 9, the sentence “112.1, 117.5, 126.7, 129.6, 131.4, 162.4 (C=O), 171.3 (C=S) ppm” should be replaced with “112.1, 117.5, 126.7, 129.6, 151.4, 162.4 (C=O), 171.3 (C=S) ppm”.
13. On page 19271, line 17, the sentence “117.3, 126.0, 129.2, 131.2, 162.4 (C=O), 171.3 (C=S) ppm” should be replaced with “117.3, 126.0, 129.2, 151.2, 162.4 (C=O), 171.3 (C=S) ppm”.
14. On page 19271, line 26, the sentence “131.3, 132.0, 162.2 (C=O), 171.1 (C=S) ppm; MS: *m*/*z* (%) = M^+^, 418 (1), 377 (5), 97 (12), 91 (100)” should be replaced with “131.3, 152.0, 162.2 (C=O), 171.1 (C=S) ppm; MS: *m*/*z* (%) = M^+^, 418 (1), 377 (5), 97 (12), 91 (100)”.
15. On page 19272, lines 7–10, the sentence “^13^C-NMR (DMSO-*d*_6_): δ = 33.46 (CH), 45.9 (CH_3_), 125.08, 127.9, 128.2, 128.9, 129.52, 131.20, 161.9 (C=O), 169.8 (C=S) ppm; MS: *m*/*z* (%) = M^+^ + 2, 422 (0.3), M^+^, 420 (1), 129 (19), 74 (39), 69 (100); Anal. calcd. for C_17_H_14_ClN_5_O_2_S_2_ (419.90): C, 48.63; H, 3.63; N, 16.68. Found: C, 49.02; H, 2.86; N, 17.01” should be replaced with “^13^C-NMR (DMSO-*d*_6_): δ = 33.46 (CH), 45.9 (CH_3_), 95.08, 127.9, 128.2, 128.9, 129.52, 131.20, 161.9 (C=O), 169.8 (C=S) ppm; MS: *m*/*z* (%) = M^+^ + 2, 422 (0.3), M^+^, 420 (1), 129 (19), 74 (39), 69 (100); Anal. calcd. for C_17_H_14_ClN_5_O_2_S_2_ (419.90): C, 48.63; H, 3.36; N, 16.68. Found: C, 49.02; H, 2.86; N, 17.01”.
16. On page 19272, lines 15–17, the sentence “^13^C-NMR (DMSO-*d*_6_): δ = 33.56 (CH), 46.33 (CH_3_), 125.24, 126.72, 127.45, 128.12, 128.43, 129.21, 129.91, 131.82, 162.40 (C=O), 171.3 (C=S) ppm” should be replaced with “^13^C-NMR (DMSO-*d*_6_): δ = 33.56 (CH), 46.33 (CH_3_), 95.24, 126.72, 127.45, 128.12, 128.43, 129.21, 129.91, 151.82, 162.40 (C=O), 171.3 (C=S) ppm”.
17. On page 19272, lines 23, 24, the sentence “^13^C-NMR (DMSO-*d*_6_): δ = 33.55 (CH), 46.41 (CH_3_), 125.27, 128.11, 128.43, 128.93, 129.86, 131.34, 161.93” should be replaced with “^13^C-NMR (DMSO-*d*_6_): δ = 33.55 (CH), 46.41 (CH_3_), 95.27, 128.11, 128.43, 128.93, 129.86, 151.34, 161.93”.
18. On page 19272, lines 32, 33, the sentence “^13^C-NMR (DMSO-*d*_6_): δ = 33.48 (CH), 45.88 (CH_3_), 125.16, 127.89, 128.16, 128.91, 129.47, 131.16, 161.89 (C=O), 169.77 (C=S) ppm” should be replaced with “^13^C-NMR (DMSO-*d*_6_): δ = 33.48 (CH), 45.88 (CH_3_), 95.16, 127.89, 128.16, 128.91, 129.47, 151.16, 161.89 (C=O), 169.77 (C=S) ppm”.
19. On page 19272, lines 37 and 38, the sentence “IR (KBr) ν_max_ (cm^−1^): 3321 (NH), 3096 (CH arom.), 2901 (CH aliph.), 1684 (C=O), 1580 (C=N), 1141, 1081 (C=S)”; should be replaced with “IR (KBr) ν_max_ (cm^−1^): 3654 (OH), 3321 (NH), 3096 (CH arom.), 2901 (CH aliph.), 1684 (C=O), 1580 (C=N), 1141, 1081 (C=S)”.
20. On page 19273, lines 2 and 3, the sentence “^13^C-NMR (DMSO-*d*_6_): δ = 33.46 (CH), 45.64 (CH_3_), 125.02, 127.84, 128.12, 128.76, 129.32, 131.14, 161.85 (C=O), 169.73 (C=S) ppm” should be replaced with “^13^C-NMR (DMSO-*d*_6_): δ = 33.46 (CH), 45.64 (CH_3_), 95.05, 115.02, 127.84, 128.12, 128.76, 129.32, 131.14, 151.24, 161.85 (C=O), 169.73 (C=S) ppm”.

The authors would like to apologize for any inconvenience caused to the readers by these changes which do not affect the scientific results. The manuscript will be updated and the original will remain online on the article webpage, with a reference to this Correction.
